# Northward migration of the East Asian summer monsoon northern boundary during the twenty-first century

**DOI:** 10.1038/s41598-022-13713-0

**Published:** 2022-06-16

**Authors:** Zhenqian Wang, Zhenhao Fu, Bo Liu, Zeyu Zheng, Weichen Zhang, Yangyang Liu, Fen Zhang, Qiong Zhang

**Affiliations:** 1 Key Laboratory of Western China’s Environmental Systems (Ministry of Education), College of Earth and Environmental Sciences, Lanzhou University, Lanzhou, 730000 China; 2 Department of Physical Geography and Bolin Centre for Climate Research, Stockholm University, 10691 Stockholm, Sweden; 3 College of Grassland Agriculture, Northwest A&F University, Yangling, 712100 Shaanxi China

**Keywords:** Atmospheric science, Climate change

## Abstract

The northern fringe area of the East Asian summer monsoon (EASM) between arid and semiarid regions is a fragile eco-environment zone and ecological transition zone, and it is highly sensitive to climate change. Predicting the future migration of the northern boundary of the EASM is important for understanding future East Asian climate change and formulating of decisions on ecological protection and economic development in arid and semiarid regions. The reanalysis dataset and simulations of 23 models from the Coupled Models Intercomparison Project Phase 6 (CMIP6) were used to investigate the response of the boundary of the ESAM to the global warming. The multi-model ensemble showed a northwestward migration of the EASM northern boundary during the near-term (2020–2060) and late-term (2061–2099) of the twenty-first century under various Shared Socioeconomic Pathways (SSPs). The northern boundary migrated northwestward by 23–28 and 74–76 km in the near-term and late-term respectively, under SSP1-2.6, 2-4.5 and 3-7.0 and by ~ 44 km and ~ 107 km respectively during the near-term and late-term under SSP5-8.5. During the twenty-first century, under various SSPs, the surface of the East Asian subcontinent warmed more than the ocean, thereby increasing the contrast of near-surface temperature and sea level pressure in summer between the East Asian subcontinent and the surrounding oceans. In turn, the intensified land–sea thermal contrast reinforced the EASM meridional circulation and thus transported more moisture from the Indian Ocean into northern China. Additionally, a poleward migration and weakening of the East Asian subtropical westerly jet would also favor an increase in precipitation, eventually caused a northwestward migration of the EASM northern boundary. The results suggest that the arid and semiarid ecotone will become wetter, which could dramatically improve the eco-environment in the future.

## Introduction

East Asia is a monsoon climate dominated region, and the low-level prevailing wind in East Asia is seasonally reversed from southerly in summer to northerly in winter. In summer, the lower troposphere southerly winds transport abundant water vapor from the tropical oceans to East Asia, thus inciting the rainy season in East Asia^1–4^. The East Asian summer monsoon (EASM) is the most active and influential circulation system, which brings more than half of the annual precipitation to East Asia^5,6^, influencing nearly one-fifth of the world’s population living in eastern China, Japan, and the Korean peninsula. The precipitation associated with EASM circulation has far-reaching influences on the local ecosystem, agriculture and industry through its regulation of the available water resources^7,8^.

The EASM northern boundary is the northernmost position EASM can reach. The northern boundary is not static and exhibits significant interannual fluctuations following the variation of EASM. The range of the fluctuation thus form the monsoon transition zone^9,10^. The monsoon transition zone is a narrow belt-shaped region with a northeast-southwest direction. It is a transition zone between not only monsoon and non-monsoon regions but also arid and semiarid regions. The annual mean precipitation in the monsoon transition zone is often at a critical threshold for ecological maintenance and crop growth, resulting in a high sensitivity to climate change and associated natural hazards^11^. The EASM northern boundary is closely related to the location of the monsoon rain belt, and many natural disasters, especially the droughts and floods, are associated with the change of the northern boundary of the EASM^12^. The southward retreat of the EASM northern boundary usually results in droughts caused by insufficient summer rainfall in northern China^13^, which has negative impacts on the development of the local ecosystem and food security.

Due to its importance and climatic sensitivity, a great deal of effort has been devoted to investigating the characteristics of the northern boundary of the EASM, including its definitions^9,14–16^, fluctuations^17–20^ and influence on the surrounding climate^16,21^. Previous studies have investigated the advancing and retreating of the EASM northern boundary by using different climate variables, such as precipitation^9,16,17,22^, pseudoequivalent potential temperature^23^ or dewpoint temperature^24^. However, uncertainties still remain. First, the movement direction of the EASM northern boundary is inconsistent. For example, the EASM northern boundary has gradually migrated equatorward with the weakened EASM during the past decades, which is attributed by several studies to the meridional inhomogeneity of surface warming over the northern East Asia due to global warming^25,26^. In contrast, other studies have suggested an enhanced EASM and poleward-migrated EASM northern boundary due to global warming^17–19,22,27^. Second, although the EASM northern boundary migrated meridionally under the global warming climate, the mechanism differs in different warm periods. For example, in the recent decades, the meridional inhomogeneity of surface warming over northern East Asia under global warming has led to a reduction in local atmospheric baroclinicity, and thus suppressed extratropical cyclone activity over Mongolia, resulting in a southward withdrawal of the EASM northern boundary^25^. Previous studies on past warm climate show that the changes in external forcing, for instance, the increased solar radiation during the mid-Holocene^18^, or increased greenhouse gases in the atmosphere during mid-Pliocene^17^, both can enhance the land-sea contrast and strengthen the western North Pacific subtropical high (WNPSH), lead to the northward movement of the EASM northern boundary^17,18^. A few other studies suggest that northward movement of the westerly jet and northwestward extension of WPSH together modulate the northward movement of the EASM northern boundary^10,19^.

The Sixth Assessment Report of the Intergovernmental Panel on Climate Change (IPCC) indicated that human influence has warmed the climate at a rate that is unprecedented in the past 2000 years and that climate change has exerted significant influences on bio-geophysical systems^28^. Although climate warming is projected to continue, future climate changes will be uneven spatially. Predicting future changes in the EASM northern boundary migration is critical for the climate mitigation and adaptation in the EASM region.

Earth system models have become an essential tool for understanding, simulating and predicting monsoon variations. A recent series of future climate projection experiments under Shared Socioeconomic Pathways (SSPs) scenarios^29^ from the latest and state-of-the-art climate models have been released by the Coupled Model Intercomparison Project Phase 6 (CMIP6), providing us with the opportunity to examine the future changes in the EASM northern boundary and EASM circulation. In this work, we employed the model outputs of 115 experiments from 23 coupled ocean–atmosphere general circulation models from CMIP6^30^ to assess future changes in the EASM northern boundary and EASM circulation under future SSP scenarios.

## Materials and methods

### Observational and model data

To evaluate the capabilities of the models, the Global Precipitation Climatology Project (GPCP) Version 2.3 Combined Precipitation dataset^31^ was obtained, which describes monthly precipitation from 1981 to 2010. The global geopotential height, wind, and temperature from 1981 to 2010 were obtained from the National Centers for Environmental Prediction and National Center for Atmospheric Research (NCEP/NCAR) Reanalysis 1 dataset^32^. The horizontal resolution of the above two datasets is 2.5° × 2.5°.

We analyzed the total 115 simulations from the 23 CMIP6 models, which included Historical and the Shared Socioeconomic Pathways (SSPs) 1-2.6 (+ 2.6 W m^2^ imbalance; low forcing sustainability pathway; SSP1-2.6), 2-4.5 (+ 4.5 W m^2^; medium forcing middle-of-the-road pathway; SSP2-4.5), 3-7.0 (+ 7.0 W m^2^; medium- to high-end forcing pathway; SSP3-7.0), 5-8.5 (+ 8.5 W m^2^; high-end forcing pathway; SSP5-8.5)^29^. Details of the CMIP6 models are listed in Table 1 and documented on the Coupled Model Intercomparison Project website (https://esgf-node.llnl.gov/projects/cmip6/).

**Table 1:** Description of CMIP6 models used in this study.

Model	Institute	Horizontal Resolution
ACCESS-CM2	Australian Community Climate and Earth System Simulator	144 × 192
ACCESS-ESM1-5	Australian Community Climate and Earth System Simulator	145 × 192
AWI-CM-1-1-MR	Alfred Wegener Institute	192 × 384
BCC-CSM2-MR	Beijing Climate center, China Meteorological Administration	160 × 320
CAS-ESM2-0	Chinese Academy of Sciences	128 × 256
CAMS-CSM1-0	Chinese Academy of Meteorological Sciences	160 × 320
CESM2-WACCM	National Center for Atmospheric Research	192 × 288
CMCC-CM2-SR5	Fondazione Centro Euro-Mediterraneo sui Cambiamenti Climatici	192 × 288
CMCC-ESM2	Fondazione Centro Euro-Mediterraneo sui Cambiamenti Climatici	192 × 288
EC-Earth3-Veg	European Research Consortium	256 × 512
FGOALS-f3-L	Chinese Academy of Sciences	180 × 288
FGOALS-g3	Chinese Academy of Sciences	80 × 180
GFDL-ESM4	Geophysical Fluid Dynamics Laboratory	180 × 288
INM-CM4-8	Institute of Numerical Mathematics of the Russian Academy of Sciences	120 × 180
INM-CM5-0	Institute of Numerical Mathematics of the Russian Academy of Sciences	120 × 180
IPSL-CM6A-LR	Institution Pierre-Simon Laplace	143 × 144
MIROC6	Japan Agency for Marine-Earth Science and Technology	128 × 256
MPI-ESM1-2-HR	Max Planck Institute for Meteorology	192 × 384
MPI-ESM1-2-LR	Max Planck Institute for Meteorology	96 × 192
MRI-ESM2-0	Meteorological Research Institute	160 × 320
NorESM2-LM	Bjerknes Centre for Climate Research	96 × 144
NorESM2-MM	Bjerknes Centre for Climate Research	192 × 288
TaiESM1	Research Center for Environmental Changes, Academia Sinica, Taiwan	192 × 288

### Evaluation of the models

**Figure 1:**
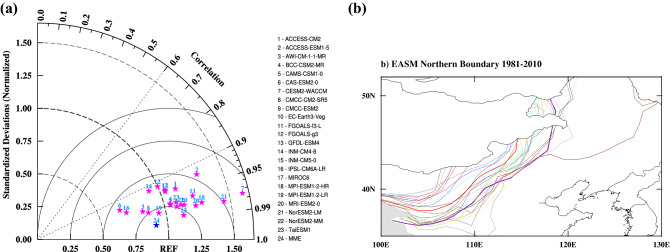
(**a**) Taylor diagram showing the pattern correlation coefficient and normalized standardized deviations in the present day (1981–2010) simulation of the climatological summer precipitation over East Asia (4°N-53°N, 73°E-150°E) of CMIP6 models. (**b**) Climatological distribution of the EASM northern boundary over eastern China for 1981–2010 by using multi-model ensemble (MME, red thick line), bias-corrected MME (blue thick line), GPCP (pink dash line) and 23 CMIP6 model outputs (other color thin lines) data. Maps were generated using the NCAR Command Language (The NCAR Command Language (Version 6.6.2) [Software]. (2019). Boulder, Colorado: UCAR/NCAR/CISL/TDD. https://www.ncl.ucar.edu/Download/).

In this study, we defined the EASM northern boundary by using the 2 mm/day isohyet in summer (May–September; MJJAS)^9^, and the Taylor diagram^33^ is applied to quantitatively evaluate the skill of CMIP6 in reproducing the present day climatological summer precipitation over east Asia (Fig. 1a).

Most (more than two thirds) of the CMIP6 models simulated larger standard deviations (SDs) of the precipitation than the observation, and the CMIP6 multi-model ensemble mean (MME) shows a slightly smaller SD relative to the observations. In terms of spatial correlation, the correlation coefficient between the CMIP6 MME and observations is 0.993. The Taylor diagram indicates that the CMIP6 MME could convincingly capture the present day climatological summer precipitation over East Asia.

The MME method is commonly applied to suppress model drift and internal variability and improve the reliability of climate model prediction^34^, but this method has common background bias in ensemble models, which leads to bias in MME changes and limits the reliability of climate predictions. As shown in Fig. 1b, the MME method smooths the EASM northern boundary, which makes it more reliable than that described by individual model. However, this method leads to more northerly migration of the EASM northern boundary than that in the observation. Therefore, the method of linear correction was used to reduce the uncertainties of future climate prediction^35,36^. All data were remapped to a common regular 1.25° × 1.25° grid using bilinear interpolation remapping, and all simulation data were bias-corrected.

The following method was used to deal with systemic biases in simulated. Specifically, the precipitation was bias-corrected according to

1 $$\left[ {{\text{P}}_{{{\text{adj}}}} } \right]_{{{\text{Y}},{\text{ M}}}} = \, \left[ {\text{P}} \right]_{{{\text{Y}},{\text{M}}}} \times \, \left( {\left[ {{\text{P}}_{{{\text{obs}},{\text{clim}}}} } \right]_{{\text{M}}} + \, 0.0{1}} \right)/\left( {\left[ {{\text{P}}_{{{\text{clim}}}} } \right]_{{\text{M}}} + \, 0.0{1}} \right)$$

where [P]_Y,M_ and [P_adj_]_Y,M_ are the raw and bias-corrected CMIP6 historical precipitation for year Y and month M, respectively. [P_clim_]_M_ and [P_obs,clim_]_M_ are the climatology of the CMIP6 historical and GPCP precipitation for 1981–2010 on month M^20,36,37^. We added 0.01 mm per month to the denominator to avoid dividing by zero. The other variables of CMIP6 were bias-corrected according to

2 $$\left[ {{\text{V}}_{{{\text{adj}}}} } \right]_{{{\text{Y}},{\text{ M}}}} = \, \left[ {\text{V}} \right]_{{{\text{Y}},{\text{M}}}} \times \, \left[ {{\text{V}}_{{{\text{obs}},{\text{clim}}}} } \right]_{{\text{M}}} /\left[ {{\text{V}}_{{{\text{clim}}}} } \right]_{{\text{M}}}$$

where [V]_Y,M_ and [V_adj_]_Y,M_ are the original and bias-corrected CMIP6 historical climate variables for year Y and month M, respectively, and [V_clim_]_M_ and [V_obs,clim_]_M_ are the climatology of the CMIP6 Historical simulation and NECP/NCAR Reanalysis 1 datasets for 1981–2010 on month M^35–37^. After the bias-corrected, even though the MME exhibits almost same position to the observation (Fig. 1b), this method does not completely reduce the uncertainties in the climate simulations.

## Results

### Spatial evolution of the EASM northern boundary

**Figure 2:**
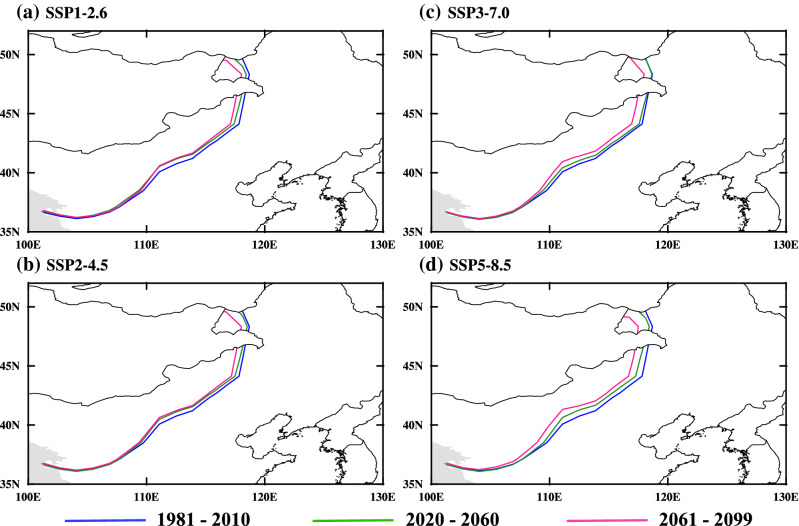
Climatological distribution of the EASM northern boundary over eastern China projected by CMIP6 MME. The solid lines in (**a**–**d**) indicate the boundaries for 2020–2060 (near-term) and 2061–2099 (late-term) in SSP1-2.6 (**a**), SSP2-4.5 (**b**), SSP3-7.0 (**c**), and SSP5-8.5 (**d**) during the twenty-first century compared to the present day. Maps were generated using the NCAR Command Language (The NCAR Command Language (Version 6.6.2) [Software]. (2019). Boulder, Colorado: UCAR/NCAR/CISL/TDD. https://www.ncl.ucar.edu/Download/).

In this study, the EASM northern boundary is measured by a 2 mm/day isohyet in summer (May–September, MJJAS), and this boundary index has a good capability for describing the northern boundary of the EASM and capturing the variations in summer monsoon circulation^9^. The changes of EASM northern boundary are gradually evident from western to eastern China, the most obvious northwestward migration of the EASM northern boundary is between 110° E and 120° E. It migrates ~ 27 km (SSP1-2.6, Fig. 2a), ~ 28 km (SSP2-4.5, Fig. 2b), ~ 23 km (SSP3-7.0, Fig. 2c), and ~ 44 km (SSP5-8.5, Fig. 2d) during the near-term (2020–2060). During the late-term (2061–2099), the EASM northern boundary migrates ~ 75 km (SSP1-2.6, Fig. 2a), ~ 76 km (SSP2-4.5, Fig. 2b), ~ 74 km (SSP3-7.0, Fig. 2c), and ~ 107 km (SSP5-8.5, Fig. 2d) (summarized in Table 2). The EASM northern boundary generally exhibits a northwestward migration during the twenty-first century compared to that in the present day. In the near-term or late-term future, the largest northwestward migration of the EASM northern boundary occurs under the high-end forcing pathway (SSP5-8.5).

**Table 2:** Migration of the EASM northern boundary.

SSPs	2020–2060	2061–2099
SSP1-2.6	~ 27 km (− 295 to 215 km)	~ 75 km (− 274 to 336 km)
SSP2-4.5	~ 28 km (− 295 to 264 km)	~ 76 km (− 260 to 264 km)
SSP3-7.0	~ 23 km (− 350 to 231 km)	~ 74 km (− 275 to 249 km)
SSP5-8.5	~ 44 km (− 267 to 246 km)	~ 107 km (− 256 to 252 km)

The brackets denote the 20th and 80th percentiles of the individual models*.*

### Temporal evolution of the EASM northern boundary

**Figure 3:**
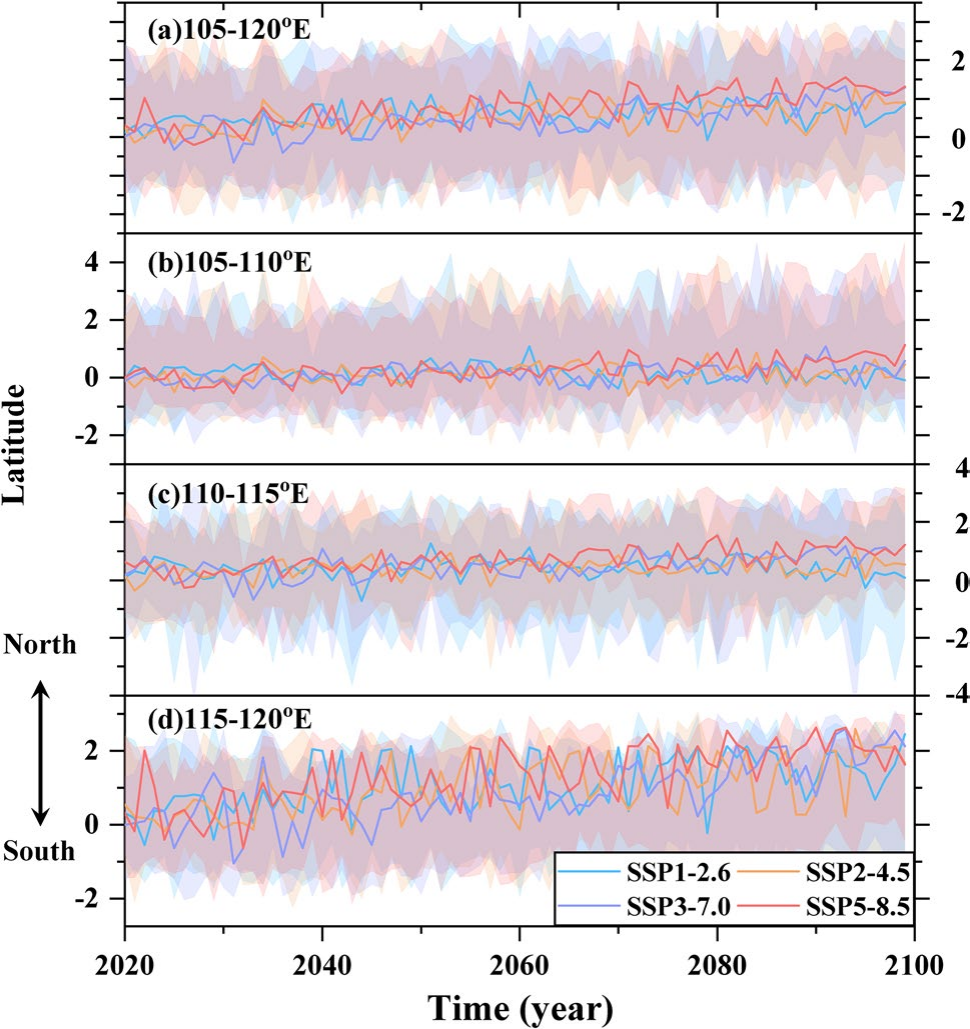
Temporal evolution of the meridional migration of the EASM northern boundary over eastern China since the twenty-first century compared to the present day within the region of (**a**) 105–120°E, (**b**) 105–110°E, (**c**) 110–115°E, (**d**) 115–120°E under SSP1-2.6 (cyan), SSP2-4.5 (orange), SSP3-7.0 (medium purple), and SSP5-8.5 (red). Solid lines indicate the meridional migration of the EASM northern boundary (unit: degree), while shadings indicate the 20th and 80th percentiles of the individual models.

According to the spatial distribution of the EASM northern boundary (Fig. 2), it has a smaller migration range in western China and a larger migration range in eastern China. Therefore, we divided the three regions to analyze the migration of the northern boundary of the EASM. Figure 3 shows the meridional migration of the EASM northern boundary in different regions during the twenty-first century compared to the present day.

Relative to the present day, a gradually poleward migration of the EASM northern boundary was found under different future emission scenarios (Fig. 3a), with differences in the amplitude of migration in different regions. In the region of 105–110°E (Fig. 3b), the EASM northern boundary displays slight fluctuations ranging from − 0.38° to 1.08°, − 0.63° to 0.88°, − 0.46° to 1.08°, and − 0.54° to 1.13° of latitude on average under SSP1-2.6, SSP2-4.5, SSP3-7.0, and SSP5-8.5, respectively. In the region of 110–115°E (Fig. 3c), the EASM northern boundary fluctuations ranging from − 0.73° to 1.27°, − 0.36° to 1.09°, − 0.68° to 1.23°, − 0.27° to 1.55° of latitude for average under SSP1-2.6, SSP2-4.5, SSP3-7.0, SSP5-8.5, respectively. In the region of 115–120°E (Fig. 3d), the EASM northern boundary exhibits strong fluctuations ranging from − 0.55° to 2.45°, − 0.27° to 2.59°, − 1.05° to 2.59°, and − 0.64° to 2.64° of latitude on average under SSP1-2.6, SSP2-4.5, SSP3-7.0, and SSP5-8.5, respectively (summarized in Table 3).

**Table 3:** The range of meridional migration of the EASM northern boundary.

SSPs	105–110°E	110–115°E	115–120°E	105–120°E
SSP1-2.6	− 0.38° to 1.08°	− 0.73° to 1.27°	− 0.55° to 2.45°	− 0.08° to 1.44°
SSP2-4.5	− 0.63° to 0.88°	− 0.36° to 1.09°	− 0.27° to 2.59°	− 0.19° to 1.28°
SSP3-7.0	− 0.46° to 1.08°	− 0.68° to 1.23°	− 1.05° to 2.59°	− 0.66° to 1.33°
SSP5-8.5	− 0.54° to 1.13°	− 0.27° to 1.55°	− 0.64° to 2.64°	− 0.20° to 1.56°

The range is the meridional migration of the EASM northern boundary since the twenty-first century relative to present day within the region of 105–110°E, 110–115°E, 115–120°E, 105–120°E under SSP1-2.6, SSP2-4.5, SSP3-7.0, and SSP5-8.5 scenarios.

In general, the meridional migration of the EASM northern boundary generally exhibits a poleward migration in eastern China under the different emission scenarios, and from the low forcing sustainability pathway (SSP1-2.6) to the high-end forcing pathway (SSP5-8.5), the magnitude of the northward migration gradually increases, and the amplitude of migration reaches its maximum under the high-end forcing pathway scenario (SSP5-8.5).

### Mechanisms of the migration of the EASM northern boundary and discussion

**Figure 4:**
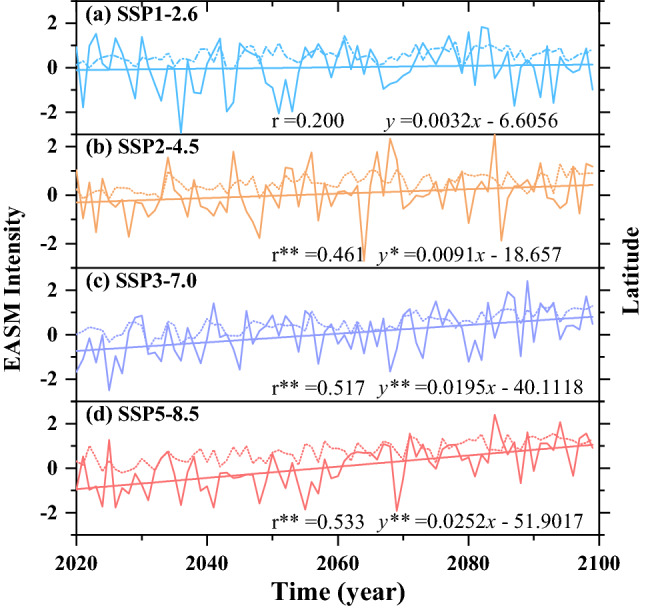
Timeseries of EASM intensity (polylines) and meridional migration of the EASM northern boundary (dotted lines) projected by CMIP6 MME during the twenty-first century. Plotted projections by CMIP6 MME for SSP1-2.6 (**a**, cyan), SSP2-4.5 (**b**, orange), SSP3-7.0 (**c**, medium purple), and SSP5-8.5 (**d**, red) compared to the present day. The solid lines indicate the linear fit. r is the correlation coefficient between EASM intensity and meridional migration of the EASM northern boundary. The asterisk indicates a 5% of significance level and the double asterisk indicate a 1% of significance level.

Previous studies suggested that the EASM circulation system largely contributes to the changes in precipitation in northern China^6,18,38,39^. Herein, we focus on the impacts of the global warming on the EASM circulation system and northward migration of the EASM northern boundary and the possible corresponding dynamic mechanisms. EASM intensity is regionally measured by the averaged meridional wind at 850 hPa within the region of 20°–40°N and 105°–120°E^2,38,40^. The temporal evolution characteristics of the EASM are shown in Fig. 4, which indicates that EASM intensity has gradually increases with the increasing emissions relative to the present day under the different future emission scenarios. The results are consistent with the results of previous studies^38,40–44^. With the enhanced monsoon circulation, the East Asian rainband extends northward and increases the precipitation in northern China^6,19,45–47^. Corresponding to the variations in the intensity of the EASM, relative to the present day, the meridional migration of the EASM northern boundary displays consistent change trends with the intensity of the EASM (Figs. 3, 4). We further analyze the correlations between the meridional migration of the EASM northern and the EASM intensity. As shown in Fig. 4, the significant positive correlation between meridional migration of the EASM northern boundary and the EASM intensity under SSP2-4.5, SSP3-7.0, and SSP5-8.5 are indicate that the higher the emissions are, the stronger the EASM, and the stronger northward advancing of the EASM northern boundary.

The seasonal variation in the uneven heat distribution between the sea and the land is the original driving force of the monsoon, which plays an important role in the formation and maintenance of the monsoon^4,48^. The EASM is the result of a combination of thermal contrast and topography between the Asian continent and surrounding oceans^1,48–50^. Therefore, changes in the thermal conditions of either one of the continents or the oceans may change the land–sea thermal contrast, leading to variations in the EASM.

**Figure 5:**
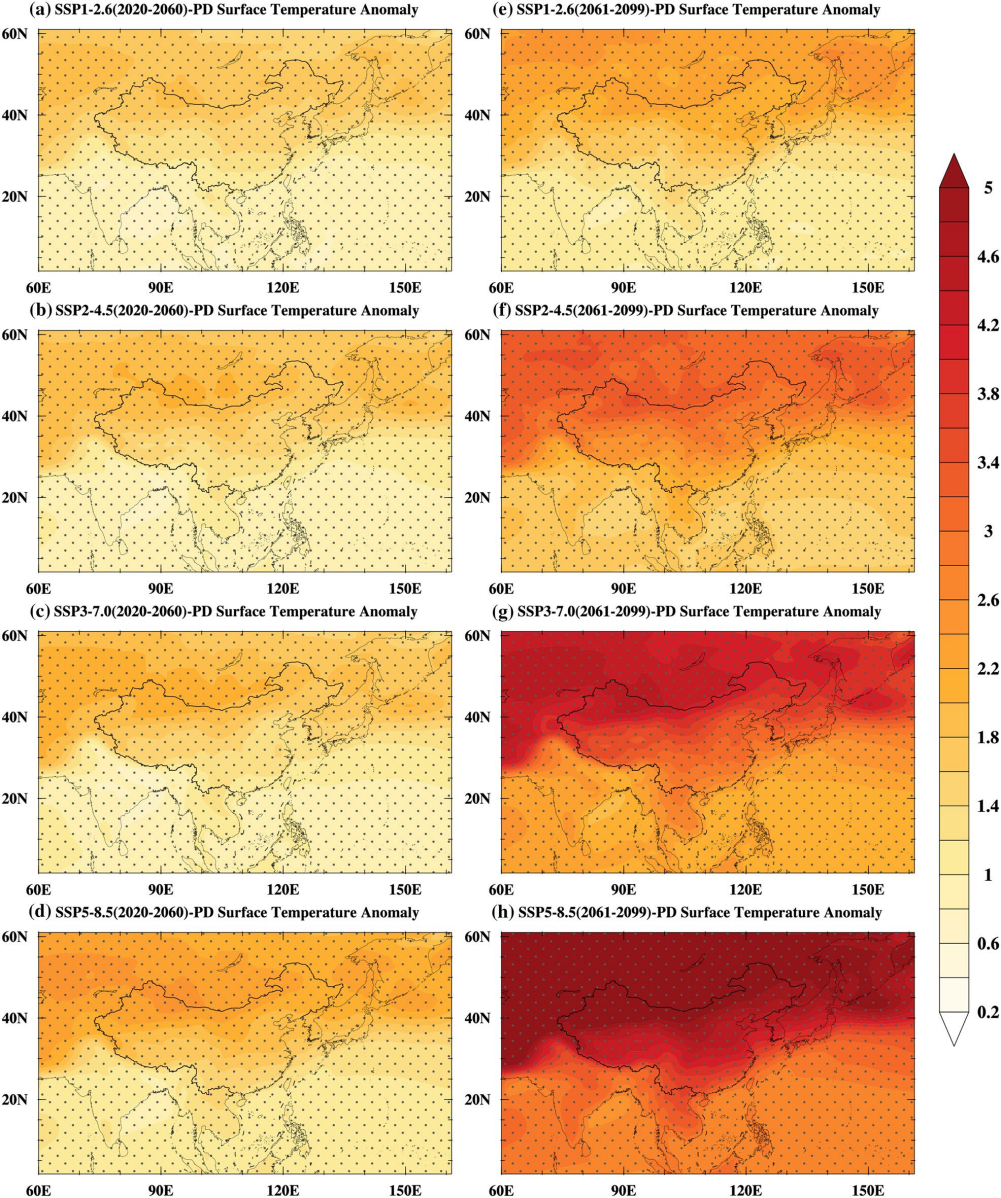
Summer surface air temperature anomalies (unit: ^o^C, shading) for 2020–2060 (near-term, **a**–**d**) and 2061–2099 (late-term, **e**–**h**) under the SSP1-2.6 (**a**,**e**), SSP2-4.5 (**b**,**f**), SSP3-7.0 (**c**,**g**), SSP5-8.5 (**d**,**h**) relative to the present day. The grey dots indicate a 5% of significance level. Maps were generated using the NCAR Command Language (The NCAR Command Language (Version 6.6.2) [Software]. (2019). Boulder, Colorado: UCAR/NCAR/CISL/TDD. https://www.ncl.ucar.edu/Download/).

To understand the dynamic mechanisms of the migration of the EASM northern boundary, the near-term and late-term surface air temperature under the SSP1-2.6, SSP2-4.5, SSP3-7.0, and SSP5-8.5 are analyzed (Fig. 5).

**Figure 6:**
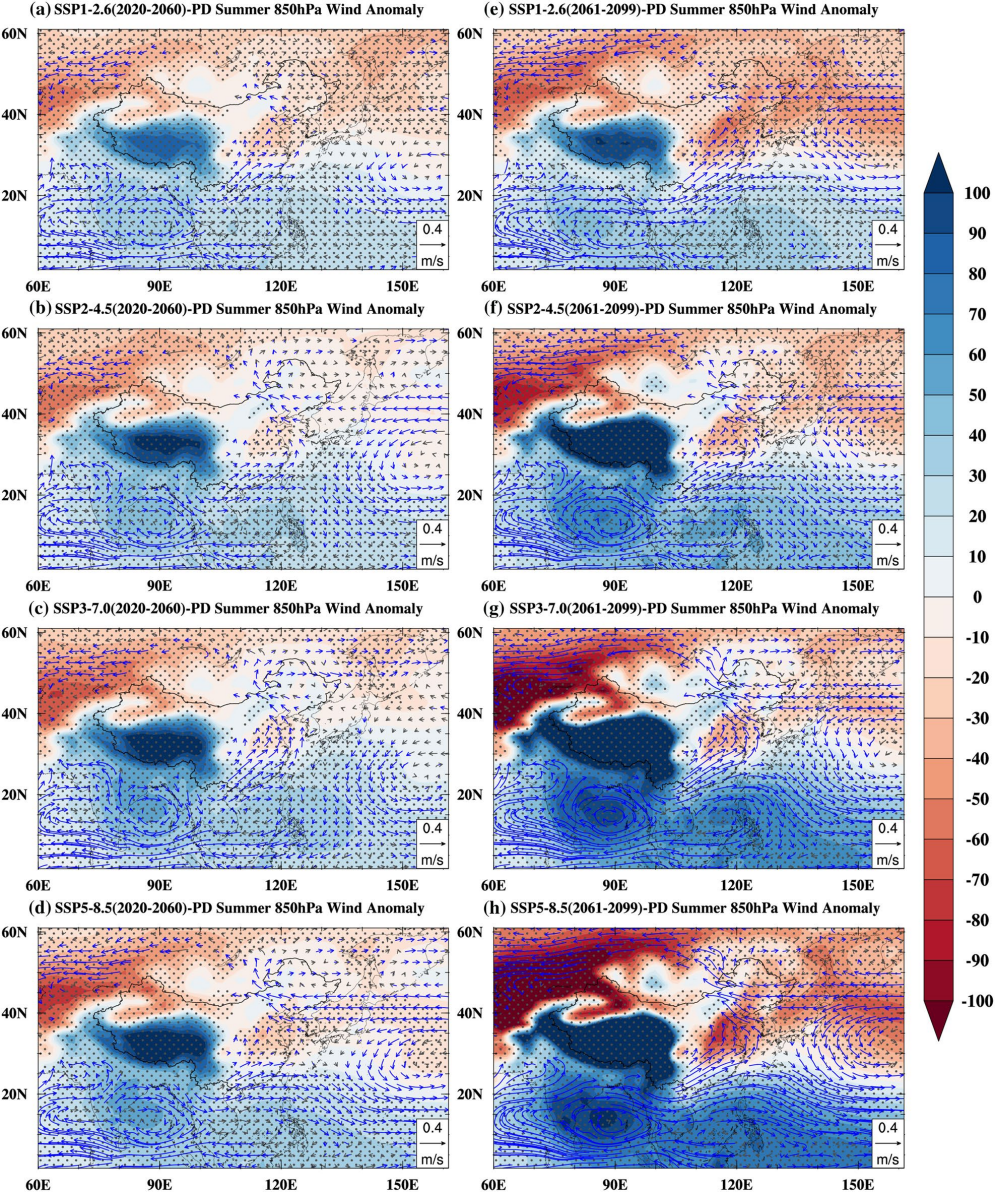
Summer sea level pressure and 850 hPa wind anomalies (unit: Pa, shading; unit: m/s, vectors) for 2020–2060 (near-term, **a**–**d**) and 2061–2099 (late-term, **e**–**h**) under the SSP1-2.6 (**a**,**e**), SSP2-4.5 (b, f), SSP3-7.0 (**c**,**g**), SSP5-8.5 (**d**,**h**) relative to the present day. The blue vectors and grey dots indicate a 5% of significance level. Maps were generated using the NCAR Command Language (The NCAR Command Language (Version 6.6.2) [Software]. (2019). Boulder, Colorado: UCAR/NCAR/CISL/TDD. https://www.ncl.ucar.edu/Download/).

**Figure 7:**
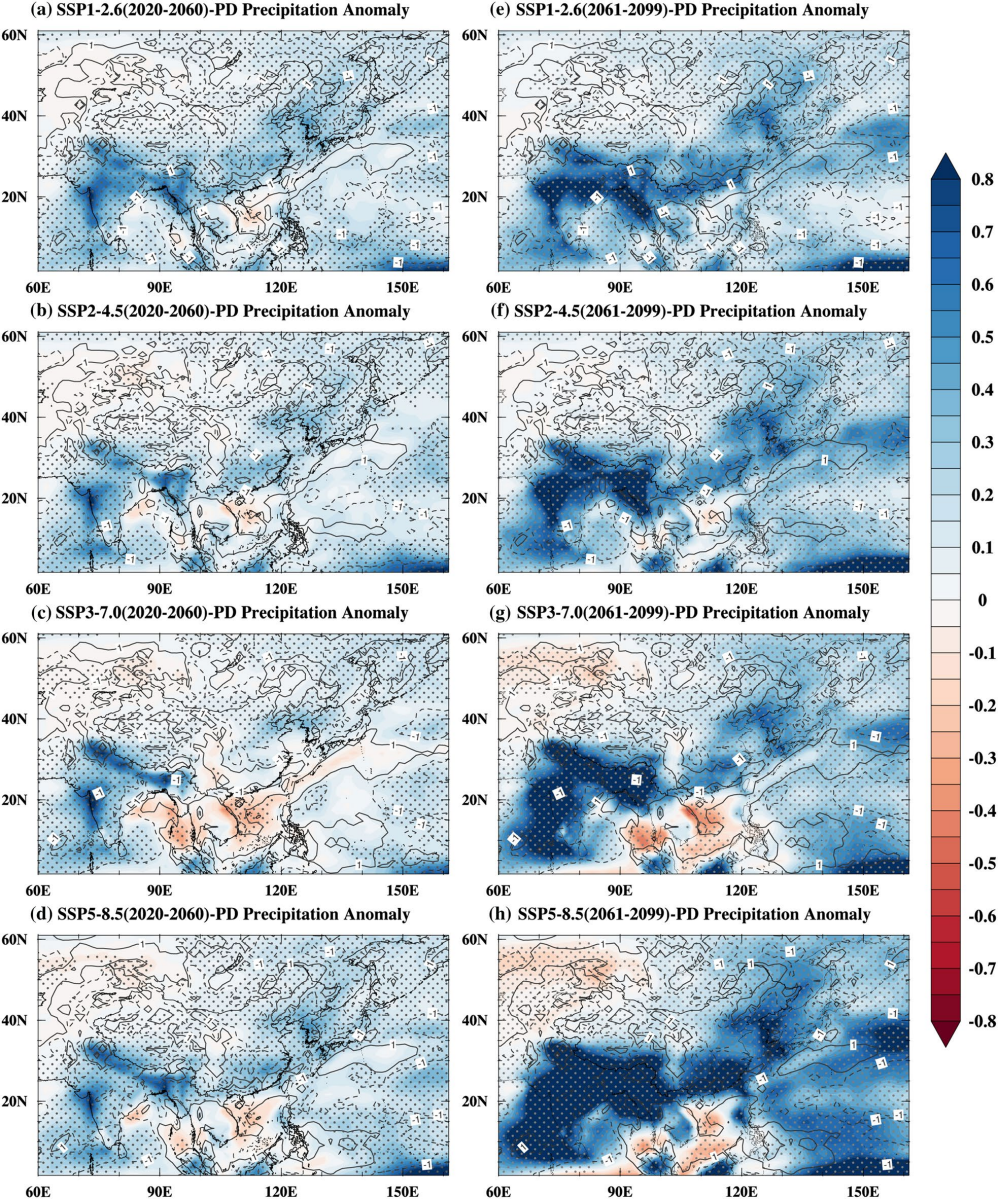
The summer precipitation anomalies (unit: mm/day, shading) together with vertical potential anomalies (unit: 10^–3^ Pa/s, dashed (solid) lines indicate upward (downward) motions) at 500 hPa for 2020–2060 (near-term, **a**–**d**) and 2061–2099 (late-term, **e**–**h**) under the SSP1-2.6 (**a**,**e**), SSP2-4.5 (**b**,**f**), SSP3-7.0 (**c**,**g**), SSP5-8.5 (**d**,**h**) relative to the present day. The grey dots indicate a 5% of significance level. Maps were generated using the NCAR Command Language (The NCAR Command Language (Version 6.6.2) [Software]. (2019). Boulder, Colorado: UCAR/NCAR/CISL/TDD. https://www.ncl.ucar.edu/Download/).

In the near-term, as the thermal inertia of the ocean is much greater than that of the land, the East Asian subcontinent exhibits stronger surface warming than its surrounding oceans due to global warming (Fig. 5a–d), thereby increasing the contrast of near-surface temperature and sea level pressure in summer between the East Asian subcontinent and its surrounding oceans (Fig. 6a–d). The enhanced meridional land–sea thermal contrast and sea-level pressure gradient enhanced the monsoon circulation and the southwesterly wind, and this enhanced southwesterly wind was beneficial for transporting the warm and moist air to northern China^20,38,40,49,51^. Additionally, the moisture transported from the north Pacific resulted from the increases in sea-level pressure gradient (Fig. 6). On the other hand, as shown in Fig. 6, anomalous high and anticyclone patterns over the Indian Ocean can facilitate the transport of moisture from the Indian Ocean to northern China^52^. The interaction between the warm and humid air transported by the southwesterly wind and the cold air transported by the mid-latitude westerly wind has caused an increase in precipitation in most parts of northern China (Fig. 7a–d). The distribution of land–sea temperature (Fig. 5e–h), sea-level pressure (Fig. 6e–h) and precipitation (Fig. 7e–h) in the late-term follow the same change patterns as in the near-term, and the amplitudes of the enhancements are much greater in the late-term.

Under global warming, the changes in temperature and circulation facilitate moisture transport to the East Asian continent and a northwestward advance of the EASM northern boundary. Therefore, under future emission scenarios, changes in land–sea temperature and circulation will induce water vapor transport to the East Asian subcontinent and northwestward migration of the northern boundary of the EASM (Fig. 2). Previous studies have investigated future variations in the EASM and associated precipitation. Recently, some studies evaluated the variations in the EASM and associated precipitation through the twenty-first century based on the multi-model results of Representative Concentration Pathway scenarios (RCPs) experiments from CMIP5 and Shared Socioeconomic Pathway scenarios experiments from CMIP6. The EASM and associated precipitation are projected to intensify over East Asia in the future under various scenarios^40–42,44,46^. In addition, the precipitation changes in East Asia were most obvious in the high emission scenarios. Figure 7 shows that the MME forecasts greater precipitation intensity over almost all of East Asia through the twenty-first century under the Shared Socioeconomic Pathway scenarios, which confirms that global warming increases EASM precipitation^40–42,44,46^.

**Figure 8:**
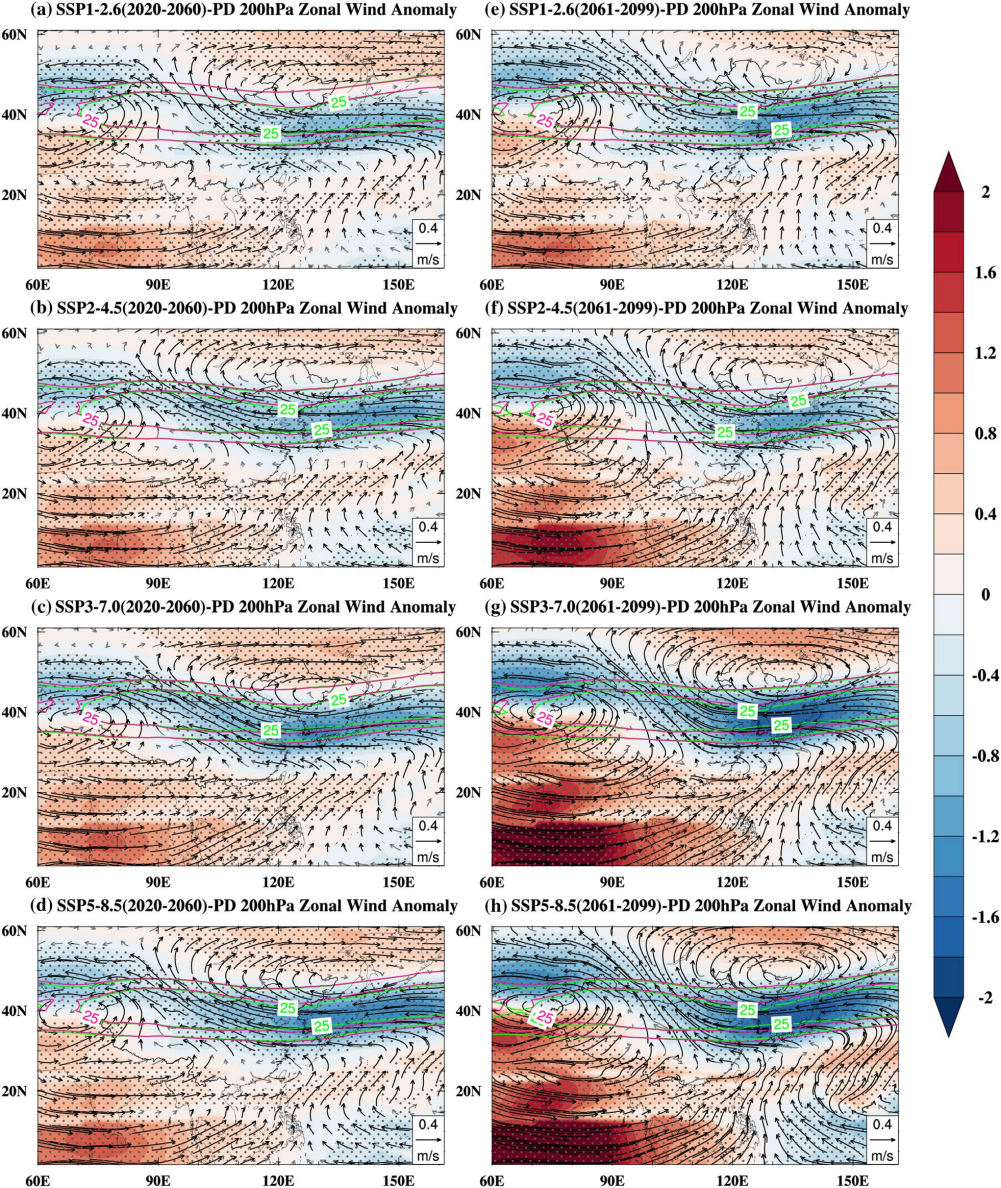
Summer 200 hPa zonal wind (unit: m/s, shading) and wind anomalies (unit: m/s, vector) for 2020–2060 (near-term, **a**–**d**) and 2061–2099 (late-term, **e**–**h**) under the SSP1-2.6 (**a**,**e**), SSP2-4.5 (**b**,**f**), SSP3-7.0 (**c**,**g**), SSP5-8.5 (**d**,**h**) relative to the present day, together with the climatologic mean of the East Asia subtropical westerly jet (unit: m/s) for the future (red contours) and the present day (green contours). The black vectors and dots indicate a 5% of significance level. Maps were generated using the NCAR Command Language (The NCAR Command Language (Version 6.6.2) [Software]. (2019). Boulder, Colorado: UCAR/NCAR/CISL/TDD. https://www.ncl.ucar.edu/Download/).

In addition to the land–sea thermal contrast, the East Asia subtropical westerly jet (EASWJ) plays a key role in the seasonal migration of the East Asian rain belt^53–55^. Previous studies confirmed that poleward EASWJ displacement causes precipitation to increase over northern China during summer^10,19,53^. Similar to the jet transition hypothesis proposed by Chiang et al.^56^, the change in the meridional position of the westerly belt modulates the precipitation in different regions of the monsoon domain. Despite a difference in amplitude between the near-term and the late-term during the twenty-first century, the spatial patterns of responses are similar in the near-term and the late-term, for conciseness, the following analyzes are based mainly on the late-term. Figure 8 indicates the anomalous patterns of EASWJ. As shown in Fig. 8, the weakening of the EASWJ under the future global warming scenarios causes the EASWJ to contract and migrate poleward. Due to the anomalous easterly wind in most of China and the anomalous westerly winds in Northeast Asia, an anomalous anticyclone appears over Northeast Asia. The poleward migration of the EASWJ and the anticyclone suggests stronger divergence in the upper troposphere, which is favorable for convection and precipitation in northern China, which is confirmed by the anomalous upward motions at 500 hPa (Fig. 7). Therefore, poleward migration of the EASWJ brings heavier precipitation over northern China, contributing to the poleward migration of the EASM northern boundary.

Notably, although the mid-Pliocene (~ 3.3–3.0 Ma) climate is regarded as an analogue for a near‐future climate scenario, larger northward migration of the EASM northern boundary was evident during the mid-Pliocene relative to the present day^17,57^. In the contrast, a smaller northward migration of the EASM northern boundary was visual in the future warming scenarios. The possible reasons for this may be as follows. First, based on the framework of the Pliocene Model Intercomparison Project (PlioMIP)^58^, this period is characterized by a similar atmospheric CO_2_ level (405 ppmv) to that of the present day, reduced land ice in Greenland and Antarctica^59^, and much more complete vegetation cover^60^. Thus, the reduced land ice in Greenland and Antarctica^61^, and the enhanced meridional land–sea thermal contrast with an anomalous anticyclone in the northwestern Pacific Ocean, are responsible for strengthening southerly winds^17^. However, in the future, anomalous cyclones in the northwestern Pacific Ocean (Fig. 6) may prevent the strengthening of southwesterly winds. Previous studies have attributed the northward migration of the EASM northern boundary to the enhanced WNPSH in the mid-Pliocene warm period^17^, but we noted a weak change or slight weakening of the WNPSH under future global warming scenarios^51,62^ (Fig. S1). During the mid-Pliocene, the lower temperature and less precipitation in the subtropical North Pacific reduced the latent heat in the western Pacific and intensified the WNPSH, enhanced the EASM circulation. However, these changes in WNPSH are not observed in future projections, which may explain the much less migration of the EASM northern boundary under the global warming scenarios than in the mid-Pliocene. Second, an important feature of the SSPs is that they cover a much wider range of aerosol and air pollutant emissions. Previous studies have investigated whether aerosols play an important role in driving the weakened EASM^5,50^. Therefore, aerosols may counteract the intensification of the EASM in the future. Finally, the nonlinear hydrological responses to warming in different scenarios^62,63^ can be a reason for the stronger EASM in the mid-Pliocene than in the future. This can also explain the similar fluctuations in the northern boundary of the EASM under the SSP1-2.6, SSP2-4.5 and SSP3-7.0 scenarios.

## Conclusion

By analyzing the model outputs of the CMIP6 SSPs and Historical simulations, we explored the dynamic mechanisms for the migration of the EASM northern boundary under global warming. It is projected that both the circulation and precipitation of the EASM will be strengthened through the twenty-first century under the future SSP scenarios, and the EASM northern boundary will migrate northwestward. From the low forcing sustainability pathway (SSP1-2.6) to the high-end forcing pathway (SSP5-8.5), the amplitude of migration along the EASM northern boundary gradually increases, and the amplitude of migration reaches its maximum under the high-end forcing pathway scenario (SSP5-8.5).

The enhanced land–sea thermal contrast and the poleward migration and weakened EASWJ in summer explain the substantial enhancement of the EASM circulation and northwestward migration of the EASM northern boundary under future global warming. Due to the thermal inertia of the ocean, the summertime warming of the East Asian subcontinent is much stronger than that of the surrounding oceans under future global warming. The enhanced land–sea thermal contrast enhances the EASM circulation, which helps transport the warm and moist air to northern China. In addition, the poleward migration of the EASWJ triggered upward motions in the lower troposphere, which also provided favorable conditions for the increase in precipitation in northern China, eventually caused the northwestward migration of the EASM northern boundary. These results shed light on the relationship between the migration of the EASM northern boundary and global warming, and provide useful information for the formulation of decisions on ecological protection and economic development in arid and semiarid regions.

The EASM domain is near the Pacific Ocean, which is directly and indirectly associated with the Hadley circulation and the El Niño-Southern Oscillation (ENSO) phenomenon. The possible roles of these factors in the responses of the EASM to future warming deserve further investigation.

## Supplementary Information

Supplementary Figure S1.

## Data Availability

The GPCP precipitation dataset was provided by the NOAA/OAR/ESRL PSL, Boulder, Colorado, USA, from their website at https://psl.noaa.gov/data/gridded/data.gpcp.html. The NCEP/NCAR Reanalysis 1 dataset can be downloaded from the NOAA Physical Sciences Laboratory Web site at https://www.psl.noaa.gov/data/gridded/data.ncep.reanalysis.derived.html. The CMIP6 datasets analyzed in this study are publicly available at https://esgf-node.llnl.gov/projects/cmip6/.

## References

[1] Webster PJ (1998). Monsoons: Processes, predictability, and the prospects for prediction. J. Geophys. Res-Oceans.

[2] Wang B (2002). Rainy season of the Asian-Pacific summer monsoon. J. Clim..

[3] Zhou TJ, Yu RC (2005). Atmospheric water vapor transport associated with typical anomalous summer rainfall patterns in China. J. Geophys. Res-Atmos..

[4] An ZS (2015). Global monsoon dynamics and climate change. Annu. Rev. Earth Planet. Sci..

[5] Song FF, Zhou TJ, Qian Y (2014). Responses of East Asian summer monsoon to natural and anthropogenic forcings in the 17 latest CMIP5 models. Geophys. Res. Lett..

[6] Zhang RH (2015). Changes in East Asian summer monsoon and summer rainfall over eastern China during recent decades. J. Arid Land Resour. Environ..

[7] Huang RH, Chen JL, Huang G (2007). Characteristics and variations of the East Asian monsoon system and its impacts on climate disasters in China. Adv. Atmos. Sci..

[8] Li Q, Wei FY, Li DL (2011). Interdecadal variation of East Asian summer monsoon and drought/flood distribution over eastern China in the last 159 years. J. Geogr. Sci..

[9] Chen J (2018). A climatological northern boundary index for the East Asian summer monsoon and its interannual variability. Sci. China Earth Sci..

[10] Zeng J, Zhang Q (2019). A humidity index for the summer monsoon transition zone in East Asia. Clim. Dyn..

[11] Ou TH, Qian WH (2006). Vegetation variations along the monsoon boundary zone in East Asia. Chin. J. Geophys..

[12] Shi ZT (1994). Regional characters of natural disaster in marginal monsoon belt of China. J. Arid Land Resour. Environ.

[13] Qian WH, Shan XL, Chen DL, Zhu CW, Zhu YF (2012). Droughts near the northern fringe of the East Asian summer monsoon in China during. Clim. Change.

[14] Wang AY (1999). The definition of the advance and retreat of the summer monsoon in China. Plateau Meteorol..

[15] Hu HR, Qian WH (2007). Confirmation of the north edge of East Asian summer monsoon. Prog. Nat. Sci..

[16] Huang F, Li DL, Tang X, Wang SG, Wang H (2009). Determination on the north boundary of summer monsoon in East Asian with soaking rainfall. J. Appl. Meteorol. Sci..

[17] Huang XF (2019). Northwestward migration of the northern edge of the East Asian summer monsoon during the mid-Pliocene warm period: Simulations and reconstructions. J. Geophys. Res-Atmos..

[18] Piao JL, Chen W, Wang L, Pausata FSR, Zhang Q (2020). Northward extension of the East Asian summer monsoon during the mid-Holocene. Glob. Planet. Change.

[19] Chen J (2021). Northwestward shift of the northern boundary of the East Asian summer monsoon during the mid-Holocene caused by orbital forcing and vegetation feedbacks. Quat. Sci. Rev..

[20] Wu BL, Lang XM, Jiang DB (2021). Migration of the northern boundary of the East Asian summer monsoon over the last 21,000 years. J. Geophys. Res-Atmos..

[21] Li C, Han X (2008). Relationship of northern boundary of East Asian summer monsoon and summer precipitation in eastern part of China. Plateau Meteorol..

[22] Huang XF (2021). Warming-induced northwestward migration of the Asian summer monsoon in the geological past: Evidence from climate simulations and geological reconstructions. J. Geophys. Res. Atmos..

[23] Huang S, Tang M (1987). On the structure of the Summer Monsoon regime of East Asia. J. Meteorol. Sci..

[24] Zhu QG, Yang S (1989). The northward advance and oscillation of the East-Asian Summer Monsoon. J. Nanjing Inst. Meteorol..

[25] Zhu XC (2018). A southward withdrawal of the northern edge of the East Asian summer monsoon around the early 1990s. Atmos. Ocean Sci. Lett..

[26] Zhu CW, Wang B, Qian WH, Zhang B (2012). Recent weakening of northern East Asian summer monsoon: A possible response to global warming. Geophys. Res. Lett..

[27] Yang SL (2015). Warming-induced northwestward migration of the East Asian monsoon rain belt from the Last Glacial Maximum to the mid-Holocene. Proc. Natl. Acad. Sci. USA.

[28] IPCC. *Climate Change 2021: The Physical Science Basis. Summary for Policymakers*. Sixth Assessment Report (Intergovernmental Panel on Climate Change, 2021).

[29] O'Neill BC (2016). The Scenario Model Intercomparison Project (ScenarioMIP) for CMIP6. Geosci. Model. Dev..

[30] Eyring V (2016). Overview of the Coupled Model Intercomparison Project Phase 6 (CMIP6) experimental design and organization. Geosci. Model Dev..

[31] Schneider U (2017). Evaluating the hydrological cycle over land using the newly-corrected precipitation climatology from the global precipitation climatology centre (GPCC). Atmosphere.

[32] Kalnay E (1996). The NCEP/NCAR 40-year reanalysis project. B. Am. Meteorol. Soc..

[33] Taylor KE (2001). Summarizing multiple aspects of model performance in a single diagram. J. Geophys. Res-Atmos..

[34] Gupta AS, Jourdain NC, Brown JN, Monselesan D (2013). Climate drift in the CMIP5 Models. J. Clim..

[35] Ines AVM, Hansen JW (2006). Bias correction of daily GCM rainfall for crop simulation studies. Agric. For. Meteorol..

[36] Miao CY, Su L, Sun QH, Duan QY (2016). A nonstationary bias-correction technique to remove bias in GCM simulations. J. Geophys. Res-Atmos..

[37] Feng S, Fu Q (2013). Expansion of global drylands under a warming climate. Atmos. Chem. Phys..

[38] Li ZB, Sun Y, Li T, Ding YH, Hu T (2019). Future changes in East Asian summer monsoon circulation and precipitation under 1.5 to 5 degrees C of warming. Earths Future.

[39] Li J, Wang B, Yang YM (2020). Diagnostic metrics for evaluating model simulations of the East Asian monsoon. J. Clim..

[40] Jiang DB, Tian ZP (2013). East Asian monsoon change for the 21st century: Results of CMIP3 and CMIP5 models. Chin. Sci. Bull..

[41] Bao Q (2012). Projected changes in Asian summer monsoon in RCP scenarios of CMIP5. Atmos. Ocean Sci. Lett..

[42] Kitoh A (2017). The Asian monsoon and its future change in climate models: A review. J. Meteorol. Soc. Jpn..

[43] Wang B, Jin CH, Liu J (2020). Understanding future change of global monsoons projected by CMIP6 models. J. Clim..

[44] Chen ZM (2020). Global land monsoon precipitation changes in CMIP6 projections. Geophys. Res. Lett..

[45] Wang L, Chen W (2014). A CMIP5 multimodel projection of future temperature, precipitation, and climatological drought in China. Int. J. Climatol..

[46] Liu YY, Li Y, Ding YH (2020). East Asian summer rainfall projection and uncertainty under a global warming scenario. Int. J. Climatol..

[47] Goldsmith Y (2017). Northward extent of East Asian monsoon covaries with intensity on orbital and millennial timescales. Proc. Natl. Acad. Sci..

[48] Halley E (1686). An historical account of the trade winds, and monsoons, observable in the seas between and near the Tropicks, with an attempt to assign the physical cause of the said winds. Philos. Trans. R. Soc..

[49] Kamae Y, Watanabe M, Kimoto M, Shiogama H (2014). Summertime land-sea thermal contrast and atmospheric circulation over East Asia in a warming climate-Part I: Past changes and future projections. Clim. Dyn..

[50] Li ZQ (2016). Aerosol and monsoon climate interactions over Asia. Rev. Geophys..

[51] He C, Zhou W (2020). Different enhancement of the East Asian summer monsoon under global warming and interglacial epochs simulated by CMIP6 models: Role of the subtropical high. J. Clim..

[52] Fang KY, Chen DL, Li JB, Seppa H (2014). Covarying hydroclimate patterns between monsoonal Asia and North America over the past 600 years. J. Clim..

[53] Liang XZ, Wang WC (1998). Associations between China monsoon rainfall and tropospheric jets. Q. J. R. Meteorol. Soc..

[54] Wang SX, Zuo HC, Zhao SM, Zhang JK, Lu S (2018). How East Asian westerly jet's meridional position affects the summer rainfall in Yangtze-Huaihe River Valley?. Clim. Dyn..

[55] Herzschuh U (2019). Position and orientation of the westerly jet determined Holocene rainfall patterns in China. Nat. Commun..

[56] Chiang JCH (2015). Role of seasonal transitions and westerly jets in East Asian paleoclimate. Q. Sci. Rev..

[57] Li XY, Jiang DB, Tian ZP, Yang YB (2018). Mid-Pliocene global land monsoon from PlioMIP1 simulations. Palaeogeogr Palaeocl.

[58] Haywood AM (2010). Pliocene Model Intercomparison Project (PlioMIP): Experimental design and boundary conditions (Experiment 1). Geosci. Model Dev..

[59] Dolan AM (2011). Sensitivity of Pliocene ice sheets to orbital forcing. Palaeogeogr. Palaeocol..

[60] Salzmann U, Haywood AM, Lunt DJ, Valdes PJ, Hill DJ (2008). A new global biome reconstruction and data-model comparison for the Middle Pliocene. Glob. Ecol. Biogeogr..

[61] Ge JY (2013). Major changes in East Asian climate in the mid-Pliocene: Triggered by the uplift of the Tibetan Plateau or global cooling?. J. Asian Earth Sci..

[62] Li, J., Zhao, Y., Chen, D. L., Kang, Y. Z. & Wang, H. Future precipitation changes in three key sub-regions of East Asia: the roles of thermodynamics and dynamics. *Clim. Dyn.* (2021).

[63] Wu PL, Wood R, Ridley J, Lowe J (2010). Temporary acceleration of the hydrological cycle in response to a CO_2_ rampdown. Geophys. Res. Lett..

