# Supplementary material for: Northward migration of the East Asian summer monsoon northern boundary during the twenty-first century
Source: Sci Rep. 2022 Jun 16;12:10066. doi: 10.1038/s41598-022-13713-0 (PMC9203458; doi:10.1038/s41598-022-13713-0)

Figure S1 The summer precipitation anomalies (unit: mm/day, shading), together with the 0m eddy geopotential height for the future (pink contours, SSP1-2.6 (a, e), SSP2-4.5 (b, f), SSP3-7.0 (c, g), SSP5-8.5 (d, h)) and the present day (blue contour). The grey dots indicate a 5% of significance level. Maps were generated using the NCAR Command Language (The NCAR Command Language (Version 6.6.2) [Software]. (2019). Boulder, Colorado: UCAR/NCAR/CISL/TDD. <https://www.ncl.ucar.edu/Download/>).


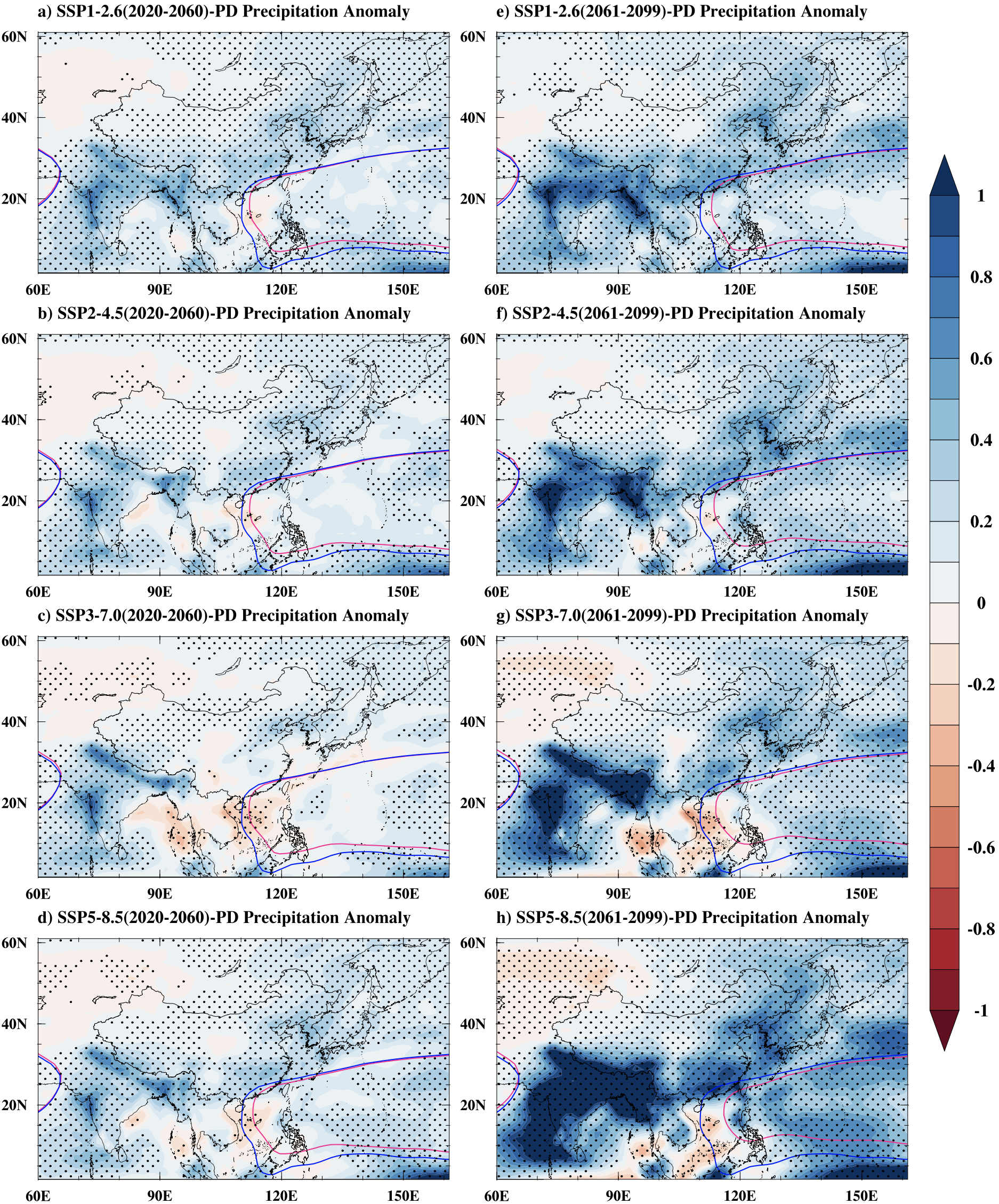

Supplement: Supplementary file 1 — Supplementary Figure S1. [file 41598_2022_13713_MOESM1_ESM.docx]
